# Oxidative Stress and Microglial Cells in Parkinson's Disease

**DOI:** 10.1155/2012/401264

**Published:** 2012-03-22

**Authors:** Lynda J. Peterson, Patrick M. Flood

**Affiliations:** North Carolina Oral Health Institute, The University of North Carolina at Chapel Hill, CB#7454, Chapel Hill, NC 27599-7454, USA

## Abstract

Significant evidence has now been accumulated that microglial cells play a central role in the degeneration of DA neurons in animal models of PD. The oxidative stress response by microglial cells, most notably the activity of the enzyme NADPH oxidase, appears to play a central role in the pathology of PD. This oxidative stress response occurs in microglia through the activation of the ERK signaling pathway by proinflammatory stimuli, leading to the phosphorylation and translocation of the p47^phox^ and p67^phox^ cytosolic subunits, the activation of membrane-bound PHOX, and the production of ROS. Therapeutic anti-inflammatories which prevent DA neurodegeneration in PD, including anti-inflammatory cytokines, morphinan compounds, NADPH oxidase inhibitors, NF-**κ**B inhibitors, and **β**2-AR agonists, all function to inhibit the activation of the PHOX in microglial cells. These observations suggest a central role for the oxidative stress response in microglial cells as a mediator or regulator of DA neurodegeneration in PD.

## 1. Neurodegeneration and Inflammation in Parkinson's Disease

Parkinson's disease (PD) is a progressive degenerative disorder of the central nervous system (CNS) that leads to impairment of motor skills and speech, as well as other functions [1]. In the United States, it is estimated that between 0.1-0.2% of all individuals are at some stage in the progression of Parkinson's Disease. This rate is somewhat higher in the elderly and those experiencing head trauma. There are several different forms of Parkinson's disease, with the most common being either the familial or the idiopathic forms. The familial forms result from alterations in genes such as *α*-synuclein, LRRK2, parkin, PINK-1, or DJ-1 [2–7], while the idiopathic forms arise from environmental factors such as exposure to toxins, head trauma, infection, or unknown etiology [8–13]. While onset before age 30 is rare, up to 10% of cases of idiopathic PD begin by age 50 [14]. The primary symptoms of PD are caused by the insufficient formation and action of dopamine, and they are manifest by decreased stimulation of the motor cortex by the basal ganglia. The loss of dopamine, which is predominantly produced by the DA neurons within the substantia nigra (SN) region and striatum of the brain, results in tremors, muscle rigidity, cognitive dysfunction, changes in speech patterns, muscle rigidity, and a dynamic progressive dysfunction that can lead to a total loss of all physical movement and ultimately death [7, 15–18].

Although the disease mechanisms that ultimately cause PD are still not fully understood, it is believed that the progressive nature of Parkinson's disease is characterized by chronic inflammation-induced neurodegeneration of dopamine-producing neurons within the SN and striatum [13, 19–23]. It is now well documented that microglial activation results in the loss of dopaminergic neurons (DA-neurons) in patients with PD [24–28]. Additionally, levels of proinflammatory cytokines, including TNF*α*, IL-1*β*, IL-6, as well as reactive oxygen species (ROS) are elevated in the brains and peripheral blood mononuclear cells (PBMCs) of patients with PD [29–31]. Nitrite in the cerebrospinal fluid as well as increased expression of inducible nitric oxide synthase (iNOS) within the SN has also been found in PD patients [32, 33], contributing to strong evidence of oxidative stress in the SN of PD patients [25, 34]. These observations, taken together suggest that progressive PD is driven by the inflammatory response of microglial cells, and that the action of these cells, including oxidative stress responses and ROS production, may play a key role in the neurodegeneration seen in PD. 

## 2. Microglial Cells and Oxidative Stress in Parkinson's Disease

The etiology of idiopathic PD now suggests that chronic production of inflammatory mediators by microglial cells, [31, 32, 35], particularly the generation of ROS and NO by activated microglia [23, 33, 34, 36, 37], mediates the majority of DA-neuronal tissue destruction [38]. Microglia are the resident macrophages of the brain and as such play critical roles in the development and maintenance of the neural environment [39]. Although microglia continually survey the surrounding tissue, they remain in essentially a quiescent state under tight regulation until they become activated in response to perturbations in the brain's microenvironment or changes in the neuronal structure. Resting microglia continually “sense” conditions in the surrounding tissue by extending and contracting their cellular processes, and their encounter with extracellular triggers such as those from pathogens, structurally or genetically altered proteins, and dead or dying neuronal cells will activate a strong proinflammatory response [39, 40]. Once activated, microglia undergo morphological changes as well as phenotypic alterations in gene expression and production/activation of signaling molecules. More importantly, microglia will mount a series of responses, starting with the oxidative stress response, to produce mediators which help to eliminate the source of these proinflammatory signals. These activated microglia have dichotomous roles in neuroinflammation in that they can both mediate the inflammatory response by producing mediators which function to clear the source of the inflammatory stimuli, and they can regulate the inflammatory response by perpetuating the proinflammatory response through the continued release of inflammatory products which function to activate and regulate microglia and astrocyte responses. Conversely, when the inflammatory stimuli have been removed, microglia can function to promote neurogenesis through the release of neurotrophins and anti-inflammatory cytokines, potentially leading to neuroregeneration and wound healing within the SN and striatum [39, 41–44].

There are a number of pro-inflammatory stimuli by which microglial cells can be activated to mediate DA-neurotoxicity in rodent models of PD. Agents that directly activate microglia have been shown to lead to the loss of DA-producing neurons both *in vitro* and *in vivo*. These include LPS [37, 45, 46] and nitrated-*α*-synuclein [47]. In addition, several direct neurotoxins, such as 1-methyl-4-phenyl-1,2,3,6-tetrahydropyridine (MPTP) and 6-hydroxydopamine (6-OHDA), can activate microglia through a process called reactive microgliosis, which leads to exacerbation of DA-neuron neurotoxicity [25, 28, 48–51]. There is also a strong correlation in human patients between proinflammatory signals in the brain, including inflammation caused by traumatic brain injury [52] and infection [53, 54], and the ultimate onset of PD. Once activated, microglial cells produce a wide variety of inflammatory mediators which serve to mediate an innate immune response, including ROS and the related NO. NF-*κ*B and MAP-kinase signaling pathways control gene expression of many of these proinflammatory cytokines, chemokines, and enzymes that produce these secondary inflammatory mediators [54–57]. Our recent work, and that of others using rodent models of DA neurodegeneration, has shown that the oxidative stress response plays a central role in the etiology of PD [58–61] and that anti-inflammatory therapeutics that inhibit the oxidative stress response in microglial cells can be neuroprotective [59–61]. Furthermore, animal studies have demonstrated that the generation of ROS is an upstream event which regulates the production of other proinflammatory factors such as TNF*α* and IL-1*β* through its regulation of the transcription factor NF-*κ*B [62, 63]. Furthermore, the postmortem detection of elevated levels of ROS and NO, and evidence of oxidative-stress-mediated damage in the brain of PD patients, strongly implies microglial-induced ROS and NO involvement in the chronic degenerative process *in vivo*. Therefore, it appears that the oxidative stress response in microglial cells may be an important component of both the DA neurodegenerative response, and in the maintenance of the chronic pro-inflammatory response seen in PD patients.

Further evidence for the central role of ROS in DA neurodegeneration and PD is found in animal model systems which exhibit hyperactive microglial cells. In the normal, healthy brain, microglia are in intimate contact with neurons which maintain the microglia in the quiescent state by means of membrane-bound and secreted signaling molecules [64–66]. The most important of these suppression signals appears to be CD200 expressed on neurons and endothelial cells within the CNS and its interaction with the receptor CD200R, which is expressed on the microglia [64, 67, 68]. Interaction of CD200 and the receptor, between contacting neurons and microglia, helps to maintain the microglia in the inactive state [64, 67–71], since mice lacking expression of CD200 exhibit microglia with morphology typical of activated cells as well as heightened expression of activation markers CD11b and CD45 [69]. Microglial cells from CD200 deficient mice also release elevated levels of inflammatory mediators TNF*α*, ROS, and NO subsequent to an immune challenge [69]. A rat model of PD using mixed cultures of mesencephalic neurons and microglial cells showed that treatment with a function neutralizing antibody to CD200R enhances the susceptibility of DA neurons to neurotoxic insult [71]. Similar results were found when the mixed cultures were subjected to insult by the addition of iron in another cellular model of PD [71]. Significantly, the neutralizing antibody-treated cultures showed increased levels of NADPH oxidase-mediated superoxide production which was found to be the critical factor in DA-neuron neurotoxicity. Treatment with the superoxide inhibitors, superoxide dismutase (SOD) or catalase, attenuated the loss of DA neurons in mixed cultures treated with the anti-CD200R antibodies plus neurotoxins. Similarly, the NADPH oxidase inhibitor, apocynin, also blocked the antibody-toxin effects on DA neuron survival [71]. Impairment of CD200-CD200R interaction is functionally associated with microglial activation and the increased death of DA neurons which is induced by enhanced NADPH oxidase-mediated superoxide production as the killing factor [69, 71]. Changes in the microenvironment at the cell surface of the microglia are thus amplified into intracellular signals that result in release of ROS which then can act as both direct effectors in neurotoxicity, and as secondary signaling factors that participate in stimulation of the production of other neuroinflammatory mediators.

NADPH oxidase (PHOX) is the major enzyme for the production of extracellular superoxide in immune cells and is highly expressed in microglia. It has been found that the activation of microglia by pro-inflammatory stimuli such as lipopolysaccharide (LPS) can lead to activation of PHOX through an ERK-dependent mechanism via the phosphorylation and translocation of cytosolic components of PHOX, including the p47^phox^ and p67^phox^ cytosolic subunits of PHOX [61]. We have found that phosphorylation of Ser^345^on p47^phox^ is a prerequisite to the degeneration of DA neurons both *in vivo* and *in vitro*, and that significantly decreased DA neurotoxicity is seen in both PHOX^−/−^ mice (*in vivo*) and PHOX^−/−^ midbrain neuron/glia cultures (*in vitro*) than in PHOX^+/+^ controls [72]. Furthermore, DPI (diphenylene iodonium), a widely used NADPH oxidase inhibitor, has been found to have significant therapeutic effects in a number of inflammatory diseases, including liver disease, exothermia, and EAE [73–76], and now appears to have efficacy as a therapeutic to prevent DA neurodegeneration in PD (see below). These observations suggest that the oxidative stress response, more specifically the one mediated by NADPH oxidase, may have a central role in DA neurodegeneration.

## 3. ROS and Neuroinflammation

Reactive oxygen species (ROS) include molecules such as superoxide (O_2_^−^) and hydrogen peroxide (H_2_O_2_). In addition, H_2_O_2 _ is associated with the generation of nitric oxide (NO), another reactive species. While ROS have some essential roles in normal cell functions [77], they are more associated with their pathological effects that ultimately lead to protein and cellular damage as well as cell death [78–81]. Extracellular insults, which include inflammation, stimulate production of ROS to hyperphysiological concentrations that results in oxidative stress. Under oxidative stress, redox reactions modulate proteins and generate changes in intracellular and intercellular signaling pathways that drive changes in cellular responses [71]. Different levels of oxidative stress induce disparate consequences for cellular function including proliferation, differentiation, and cell death. At high levels of ROS production, proteins become inactivated or damaged resulting in cellular degeneration and death. In this context, redox-sensitive proteins transduce redox changes into signals that induce cellular responses. Thus, proteins can be viewed as “redox sensors” that can affect modifications in their own localization, abundance, and activities [71]. Redox-modified proteins can also affect changes (conformational, posttranslational, etc.) in other proteins as is the nature of signaling pathways [71, 82]. At nonlethal levels, the redox state of the proteins and cells can be reversible and the molecular basis of the redox changes occurs mainly on the cysteine residues. Signaling events, initiated by thiol-based redox changes on the cysteines, emanate from the protein and are transmitted to other molecules in the signaling pathway via conformational changes, post-translational modifications, and translocations. In this way, the thiol-cysteines have been called “molecular switches” and these actions have been elucidated for various components of signaling pathways including proteases, kinases, and transcription factors [71].

**Figure 1:**
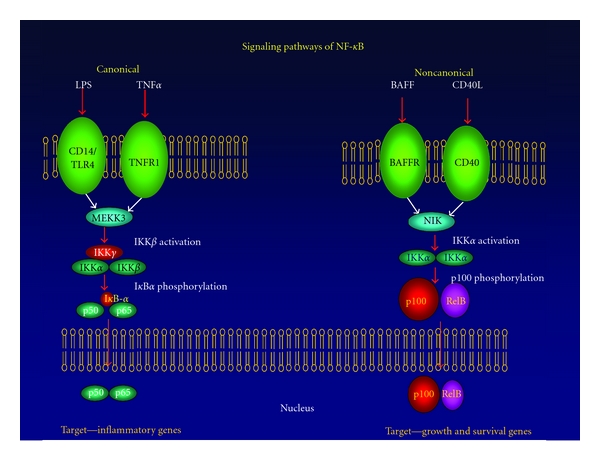
Canonical and noncanonical pathways of NF-*κ*B activation. Proinflammatory activation signals such as LPS or TNF*α* activate the canonical pathway through the activation of the IKK complex, which includes the subunits IKK*γ* (NF-*κ*B essential modulator, or NEMO), IKK*α*, and IKK*β*. This complex then phosphorylates IkB*α* (the NF-*κ*B inhibitor), which causes it to be ubiquitinated and proteolytically cleaved by the proteosome, releasing the p50/p65 heterodimer to be translocated to the nucleus, where it transcriptionally activates proinflammatory genes, including p47 and p67 of the NADPH oxidase complex. Growth and differentiation signals such as BAFF and CD40L activate the noncanonical pathway, which is initiated by NF-*κ*B inducing kinase (NIK), which phosphorylates the IKK*α* homidimer complex which does not require either IKK*γ* or IKK*β*, which subsequently phosphorylates the p100 subunit of the p100/RelB complex. The p100 protein is cleaved, which allows the p52/RelB complex to translocate to the nucleus and activate a number of growth and survival genes in target cells.

Among the transcription factors that are responsive to redox-based signaling in microglial cells is NF-*κ*B [83]. NF-*κ*B consists of two subunits which can vary according to the signaling pathway that is activated by cellular events [54, 55] (see Figure 1). Rel A (p65) and p50 are the subunits that form the heterodimer in the canonical NF-*κ*B pathway, and the p65/p50 dimer normally remains inactive in the cell's cytoplasm in complex with the I*κ*B inhibitor protein [54, 55]. When the pathway is induced by some stimuli such as oxidative stress, I*κ*B is degraded and NF-*κ*B is released to translocate to the nucleus where it binds DNA to activate transcription of genes [54, 55, 84]. It has been shown that the presence of ROS can induce activation of NF-*κ*B in the cytoplasm but can conversely inhibit NF-*κ*B activity in the nucleus [83]. Oxidation of p50 within the DNA-binding domain inhibits DNA binding thus mediating direct ROS regulation of NF-*κ*B transcriptional activity. ROS can also indirectly control NF-*κ*B-DNA binding by regulating serine phosphorylation of p65 which is necessary for RelA-DNA interaction [85]. However, ROS could both inhibit and promote NF-*κ*B activity in what has been suggested as a biphasic redox-sensitive response [86–89]. When only slight increases in ROS levels are induced in the cytoplasm, then small increases in I*κ*B degradation occur. Excessive ROS, especially when present in the nucleus, results in oxidation-mediated inhibition of NF-*κ*B-DNA binding [89]. This suggests that moderate increases in ROS induce I*κ*B degradation, leading to transcriptional activation which is usually prosurvival. Conversely, when ROS becomes excessive in the microenvironment, the nucleus becomes oxidative which inhibits NF-*κ*B transcriptional activity and abolishes prosurvival gene expression [89]. What this means for ROS and NF-*κ*B-mediated neurodegeneration is not clear since inhibition of NF-*κ*B activity or blocking ROS production has been shown to promote neuronal survival in both cellular and animal models of PD [27, 90]. Since activated microglia are a part of the clearance phase of an inflammatory response and are additionally an important source of neurotrophins in neurogeneration, microglial survival at moderate levels of ROS is crucial to their physiological function. Significantly, these cysteine-thiol-based oxidative responses to ROS are reversible suggesting signaling mechanisms similar to phosphorylation and dephosphorylation of molecular switches [89]. As with the case of phosphorylation-based signaling, ROS signaling could have stimuli-specific and cell-type-specific mechanisms. 

In phagocytes such as microglia, ROS production can be generated from several sources including intracellular peroxidases, oxidative processes in mitochondria, and NADPH oxidase activity at the cell-surface membrane [24]. Under physiological conditions, superoxide, hydroxyl radical, and hydrogen peroxide are normal byproducts of oxygen metabolism by mitochondria [77, 89]. Superoxide is generated from mitochondrial complexes I and III of the electron transport chain and can be reduced to H_2_O_2_ which is also produced by peroxisomes [89, 91]. Normally, the catalase in peroxisomes decomposes H_2_O_2_ thus preventing its toxic accumulation in the cell. Under pathological conditions, catalase activity is downregulated, H_2_O_2_ is released into the cytosol increasing oxidative stress and, in the presence of reduced metals, is converted to the hydroxyl radical. Superoxide can also react with nitric oxide to produce peroxynitrite (PN) which is an extremely active oxidizing agent [91, 92]. Excess production of reactive oxygen and nitrogen species in PD is thought to damage lipids, proteins, and DNA as shown postmortem by high levels of lipid peroxidation products, oxidized and damaged proteins, as well as oxidized DNA in the CNS of PD patients [18, 93]. PN-modified proteins have been found to accumulate in the Lewy bodies of the cells in PD brains and PN has been shown to inhibit complex-I-mediated cellular respiration [82, 91, 92, 94–96].

## 4. Successful Therapies to DA Neurodegeneration Also Target the Oxidative Stress Response

Emerging evidence demonstrates that numerous inflammatory mediators such as ROS free radicals, NO, and other products of activated immune cells can play a role in the degeneration of SN dopamine-producing neurons in several rodent models of PD [36, 79]. Therefore, it is likely that treatment with anti-inflammatory reagents directed at these proinflammatory targets could also potentially halt or slow DA neurodegeneration in these rodent models. For example, steroidal anti-inflammatory drugs (SAIDs) such as dexamethasone have been reported to show neuroprotection against MPTP- or LPS-induced toxicity in rodents [97, 98]. Nonsteroidal anti-inflammatory drugs (NSAIDS) such as aspirin and ibuprofen reduce inflammation by inhibition of COX activity and have been found to be modestly effective at slowing the progression of neurodegeneration in the MPTP rodent model [99]. Recently, strategies that block the canonical form of the proinflammatory transcription factor NF-*κ*B [27, 100], and activating the peroxisome proliferator-activated receptor-*γ* (PPAR*γ*) [101, 102], have shown beneficial effects in the modulation of inflammatory responses in mice and rats. We have used five relatively novel approaches in anti-inflammatory therapy to prevent DA neurodegeneration in rodent models of PD, and these approaches include (1) the use of endogenous anti-inflammatory cytokines IL10 or TGF*β*1, (2) the use of morphinan-related compounds; (3) the use of PHOX inhibitor DPI; (4) the targeting of IKK, a major component of the signaling pathway activating the canonical form of NF-*κ*B, which is the major transcriptional regulator of inflammation; (5) the use of *β*-2 adrenergic receptor (*β*-2AR) agonists. While none of these studies have yet been used on human patients, we will review the evidence suggesting that these successful therapies in rodents target the oxidative stress response in microglia. Furthermore, the effect of L-DOPA therapy on microglial-mediated oxidative stress responses in rodent models of DA neurodegeneration and in patients with PD will be reviewed.

## 5. Therapies Using Anti-Inflammatory Cytokines IL-10 and TGF*β*1

When ROS is the product of activated microglial cells found in the proinflammatory state, endogenous anti-inflammatory cytokines are also produced by microglial cells following elimination of the proinflammatory signal to provide a negative-feedback mechanism which converts the microglia to a wound-healing phenotype. [103, 104]. IL10 and TGF*β*1 are two major anti-inflammatory cytokines that regulate the inflammatory response by inhibiting the production of proinflammatory mediators by microglia. Studies using exogenously supplied IL10 and TGF*β*1 have shown potent effects at inhibiting the canonical signaling pathway of NF-*κ*B and in reducing neurotoxicity induced by either LPS or MPTP in PD models [60, 104–106]. We have found using an *in vitro* model of PD that the addition of IL-10 to a mixed glial cell-neuron cell culture abrogated the degeneration of the neuron cells induced by either LPS or MPTP [60], and this inhibitory effect was mediated through its inhibition of the production of extracellular superoxide in the microglia cells within the mixed cell culture [105]. These *in vitro* effects of IL-10 are confirmed by *in vivo* results from a 6-OHDA rat model of PD. In this model, sustained administration of IL10 via a viral vector significantly protected DA-neuron from death and ameliorated behavioral deficits induced by intrastriatal delivery of 6-OHDA [106]. Likewise, *in vitro* or *in vivo* studies have shown that TGF*β*1 can also protect neurons from cell death induced by oxidative stress [107] We have strong evidence that the neuroprotective effects of both IL-10 and TGF*β*1 are mainly due to their inhibition of NF-*κ*B and ROS production in microglia during initial activation or in reactive microgliosis [60, 108]. TGF*β*1 also prevents the ERK-dependent phosphorylation on p47^phox^ in the microglial cells and blocked translocation and assembly of the PHOX molecular complex to the plasma membrane. Inhibition of PHOX activation consequently reduced oxidase activities induced by LPS [108]. While the complete *in vivo* roles of IL10 and TGF*β*1 in the prevention of DA neurodegeneration in PD remain to be determined, both appear to function, at least in part, by the inhibition of oxidative stress responses in microglia.

## 6. Morphinan-Based Anti-Inflammatory Therapeutics

Several studies using PD animal models or *in vitro* cell cultures have shown that the morphinan compound dextromethorphan (DM) and its metabolites are neuroprotective due to their anti-inflammatory properties and inhibitory function towards microglia activation [59, 109, 110]. DM inhibits microglia activation and is neuroprotective when administered daily to mice that had been injected with MPTP [109]. Another metabolite of DM, 3-hydroxymorphinan (3-HM) was shown to have the greatest potency (of several tested methorphanins) for attenuating the loss of DA neurons in the SN, as well as restoring motor functions in the same MPTP-mouse model of PD [110], and clinical trials using 3-HM are currently underway in patients with progressive PD. The use of 3-HM *in vitro* protected neuronal cells in mixed glial-neuronal cells cultures by reducing MPTP-induced microgliosis and decreasing the production of ROS. [111]. Likewise, the use of other morphinan compounds, including sinomenine, a morphinan-related alkaloid compound purified from a medicinal plant (*Sinomenine acutum*) that has been traditionally used to treat inflammatory disorders [112, 113], and *d*-morphine, the dextrorotatory form of the narcotic *l*-morphine, both show strong neuroprotective effects when used both *in vitro* and *in vivo*. In all cases with these morphinan compounds, the major effect of these compounds was to inhibit microglial inflammatory responses through the inhibition of both NF-*κ*B activation and subsequently ERK phosphorylation, preventing the p47^phox^ from translocating to the plasma membrane where the complex becomes activated, and resulting in the reduction of the release of extracellular ROS [108].

## 7. Therapies using NADPH Inhibitors

Diphenyliodonium (DPI), a NADPH oxidase inhibitor, shows potent anti-inflammatory and neuroprotective effects in primary midbrain cultures, and in the LPS rodent model of PD *in vivo *[73]. Mechanistic studies revealed that DPI-elicited effects were mediated by the inhibition of LPS-induced microglial ROS production and the subsequent release of pro-inflammatory cytokine TNF*α*, and the production of nitric oxide. Further studies showed that DPI significantly reduced LPS-induced ERK phosphorylation, and the subsequent phosphorylation and translocation of the p47^phox^ cytosolic subunit [73, 108]. Taken together, our results demonstrate that DPI exerts potent anti-inflammatory and neuroprotective effects by inhibiting microglial activation through the inhibition of ERK-regulated PHOX activity.

## 8. Therapies Targeting IKK/NF-*κ*B

**Figure 2:**
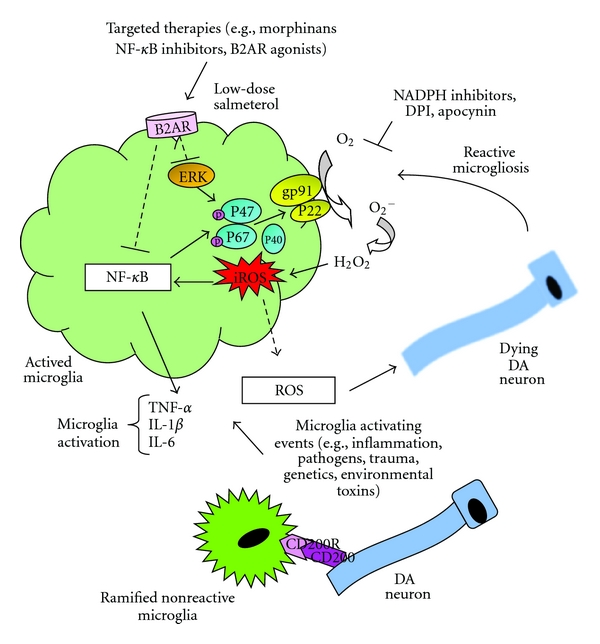
Chronic production of inflammatory mediators by microglial cells, particularly the generation of ROS and NO by activated microglia, mediates the majority of DA-neuronal degeneration and cell death. As resident brain macrophages, microglia continually survey the surrounding tissue where they remain in an inactive, quiescent state under tight regulation such as by the interaction of cell-surface molecules CD200 (expressed on neurons) and CD200R receptor on the microglia. Microglia become activated when they encounter extracellular triggers such as those from the pathogens, structurally or genetically altered proteins, toxins, and dead or dying neuronal cells. Activated microglia generate a strong proinflammatory response including the production of inflammatory cytokines (e.g., TNF*α*, IL-6, IL-1*β*) and an oxidative stress response. Most notably, the activity of the oxidative-stress enzyme, NADPH oxidase, appears to play a central role in the pathology of DA-neuron death and the progression of PD. This oxidative stress response occurs in microglia through the activation of the ERK signaling pathway by proinflammatory stimuli, leading to the phosphorylation and translocation of the p47^phox^ and p67^phox^ cytosolic subunits, the activation of membrane-bound PHOX, and the production of ROS. Therapeutic anti-inflammatories which prevent DA neurodegeneration in PD models, including anti-inflammatory cytokines, morphinan compounds, NADPH oxidase inhibitors, NF-*κ*B inhibitors, and *β*2-AR agonists, all function to inhibit the activation of the PHOX complex and ROS production in microglial cells.

Given the importance of the canonical form of transcription factor NF-*κ*B in both the production of the components of the oxidative stress response (p47 and p67 are regulated by NF-*κ*B), and in the regulatory effects of ROS in activating microglia (NF-*κ*B controls ERK activity induced by LPS signaling, Figure 2), inhibitors of the canonical pathway of NF-*κ*B should provide strong evidence of the role of oxidative stress responses in PD. Therefore, a key strategy for inhibiting neuroinflammation is to target the canonical pathway of NF-*κ*B activation without inhibiting the noncanonical pathway, which is mainly involved in cell growth, differentiation, and cell survival. In fact, specific NF-*κ*B inhibitors have recently been demonstrated to halt the progression of neurodegeneration induced by the neurotoxin MPTP in murine models of PD [27], or by activation of CNS inflammation by the intracranial injection of LPS [90]. The approach was to inhibit the canonical signaling pathway of NF-*κ*B without inhibiting the noncanonical pathway of activation by targeting the unique kinase complex of the canonical pathway called IKK (for the difference between the canonical and noncanonical signaling pathway, see Figure 1). Utilizing a peptide against IKK*γ*, which is directed against the NEMO-binding domain (NBD peptide) prior to injection of MPTP into mice significantly, inhibits the activation of NF-*κ*B within the midbrain region [27]. This inhibition of NF-*κ*B activation is accompanied by a concomitant reduction in NADPH activity and ROS production, as well as the expression of microglial cell activation marker CD11b. This protection was also seen if administration of NBD peptide was done 2 days after injection of MPTP, suggesting that NBD peptide can be used therapeutically to slow down or halt the progression of DA neurodegeneration in MPTP-treated animals [27]. 

 Likewise, use of a small molecule inhibitor of IKK*β*, called Compound A, had identical results in inhibiting ROS production from microglial cells *in vitro*, and in protecting DA neurons from LPS-induced neurodegeneration *in vitro* and *in vivo* [90]. While the specific inflammatory mediator(s) targeted by of NF-*κ*B inhibition within microglial cells remains to be determined, these data suggest that inhibition of ROS production using NF-*κ*B inhibitors may be a key component of the neuroprotection seen in PD patients [27].

## 9. Therapies Utilizing *β*2-AR Agonists

A recent family of compounds that have shown the potential to reduce inflammation and DA neurodegeneration in animal models are the long-acting *β*2-adrenergic receptor (*β*2-AR) agonists [114–120]. These *β*2-AR agonists exist in both short-acting and long-acting forms, and pharmacological studies have indicated that long-acting agonists are quite effective at inhibiting the inflammatory responses of macrophage and microglial cells. We have found that many of these long-acting agonists, including salmeterol (found in Advair) and formoterol (found in Symbicort) can inhibit the microglial oxidative stress response and inflammatory mediator production, as well as inhibiting DA neurodegeneration *in vitro *[121]. Furthermore, therapeutic administration of the long-acting *β*2-AR agonist salmeterol significantly protects DA neurons against LPS- and MPTP-induced cytotoxicity *in vivo* [121]. It is important to note that the dosages used to treat progressive neurodegeneration induced by either LPS or MPTP in mice are far below those currently used to treat patients with COPD, the current indication for this class of compounds (121), and we have previously found that exposure of macrophages and microglial cells to high or chronic doses of salmeterol can actually exacerbate rather than inhibit the inflammatory and oxidative stress response in these cells [122]. Our mechanistic studies indicate that the low-dose anti-inflammatory effects of salmeterol are mediated through the inhibition of both MAPK and NF–*κ*B signaling pathways in activated microglia, function independently of the canonical GPCR/cAMP/PKA signaling pathway, and mediate their effects only in PHOX^+^ cells. Therefore, it appears that these agonists function at least in part through the inhibition of ROS production by NADPH oxidase and add additional evidence of the central role of the oxidative stress response in microglia as a key component of DA neurodegeneration in PD. Furthermore, although no clinical or epidemiological data yet exists to suggest that the use of *β*2-AR agonists has any beneficial effect on slowing or stopping the progression of PD in human patients, our data suggests that this class of therapeutics, particularly salmeterol, may be a new and highly effective treatment to halt the progression of not only PD but also other neuroinflammatory diseases. However, given the potential for both pro- and anti-inflammatory reactions on microglial cells, therapeutic regimens using *β*2-AR agonists in the treatment of PD will need to be carefully developed and studied to determine the ultimate efficacy of these compounds.

## 10. Therapy Utilizing L-DOPA

Currently, it is believed that the progression of PD is also due to oxidative stress damage not only from microglia, but also from within the neurons themselves due in large part to increased dopamine turnover, [123, 124]. The current standard of care for the treatment of PD is the administration of L-3,4-dihydroxyphenylalanine (L-DOPA), which in the short term relieves many of the symptoms of PD but has also been found to induce an oxidative stress response in DA-neurons [125–127]. It is believed that L-DOPA at high doses actually leads to cell death in neurons due to its activation of apoptosis through the ASK1 and MAP-K signaling pathways [127, 128]. Furthermore, higher levels of markers for oxidative stress were found in patients with PD, but the origin and response of these markers to L-DOPA therapy appear to be controversial [129]. However, given the central nature of microglial cells and neuroinflammation in the pathogenesis of PD, it is important to determine the role of L-DOPA therapy on the redox reaction of microglial cells. It has been found that the oxidative stress of peripheral blood mononuclear cells (PBMCS) from patients with PD appears to be higher than healthy patients, but L-DOPA therapy actually appears to lower the redox response of these PBMCs [130]. Therefore, while L-DOPA-induced oxidative stress may play an important role in the direct toxicity of DA neurons seen in progressive PD, the effect of L-DOPA therapy on the oxidative stress response of macrophages and microglial cells and its direct damage on DA neurons is still not determined, and the anti-inflammatory effects of L-DOPA therapy may actually play a role in short-term therapy to help to protect DA-neurons from inflammatory damage mediated by activated microglial cells.
